# Editorial: Evolution of Multidrug-Resistant Clinically Important Bacteria and Fungi

**DOI:** 10.3389/fmicb.2022.910364

**Published:** 2022-05-04

**Authors:** Ying Zhu, Qiwen Yang

**Affiliations:** ^1^State Key laboratory of Complex Severe and Rare Diseases, Department of Clinical Laboratory, Peking Union Medical College Hospital, Chinese Academy of Medical Sciences, Peking Union Medical College, Beijing, China; ^2^Graduate School, Peking Union Medical College, Chinese Academy of Medical Sciences, Beijing, China

**Keywords:** evolution, multidrug resistance (MDR), bacteria, fungi, epidemiology

Due to the rapid spread of resistance and the slow development of new drugs, multidrug-resistant (MDR) bacteria and fungi have been overran issue in medical science for over 60 years (Alekshun and Levy, 2007). There are still many unanswered questions about the evolution of MDR clinically important bacteria and fungi. Monitoring of epidemiology of clinically important bacteria is beneficial as it allows for quick warning on the variation or evolution of microorganisms. The latest advance obtained from different countries and regions in the evolution of MDR in important bacteria and fungi from clinic or veterinary science is the subject of six research articles published in this Research Topic.

*Staphylococcus aureus* is an important pathogen of hospital-acquired and community-associated infections worldwide. ST59 represents the most prevalent sequence type isolated in Eastern Asia (Jin et al., 2021). McClure et al. pointed out that the origin of ST59 was disputable and they performed a detailed genetic analysis of the lineage of ST59 *Staphylococcus aureus* isolates from patients in Canada and mainland China to reveal that mobile element structure (MES) is the major difference between the two continental lineages. Analysis demonstrated the concurrent but separate evolution of North American and East Asian lineages. A more ancient MES-negative ST59 sub-lineage from North America hints at the possibility of the lineage in Eastern Asia originating from North American with acquisition of MES, and subsequently diversifying.

MDR Gram-negative bacteria is of great importance due to the transferable resistance genes, especially carbapenemase genes. Sedrakyan et al. investigated the prevalence of antimicrobial resistance (AMR), especially extended-spectrum β-lactamase (ESBL), among 291 clinical non-typhoidal *Salmonella* strains from patients in Armenia and Georgia. The most prevalent MDR serotype was ST328 *Salmonella enterica* subsp. *enterica* serovar Typhimurium (*S*. Typhimurium), most of which are *bla*_CTX−*M*−5_-positive ESBL-producers with a high-level resistance toward β-lactams. The study also encountered discordance between genome (amino acid substitutions in PBP3, *aac(6*′*)-Iaa*-harboring, AmpC) and phenotype (susceptible to β-lactam or aminoglycoside). Most studies (Card et al., 2013; Agyekum et al., 2016) have focused on the predictive value of genome to drug resistance phenotype, and few articles have reported the inconsistency between genome and phenotype, which deserves attention.

With the development of sequencing technology, research into carbapenem resistance genes has made great progress. Prevalent genotypes of pathogens and common resistance genes in different regions all over the world have been well studied. Zhao et al. explored changes in carbapenem-resistant *Klebsiella pneumoniae* (CR-KP) isolates from patients in Guangdong, China in 2016–2020. The study demonstrated that the infection rate of CR-KP has increased, and ICU and neonatal wards have become the key infection areas. ST 11 type harboring serine enzyme caused by *bla*_KPC_ are the most predominant CR-KP. Polygenic strains and novel ST11 *ropB*-mutation and *infB*-mutation have been discovered, which suggest clinical treatment difficulty. Singh et al. performed meta-analyses of the association between the isolation of ESBL from human clinical and non-clinical specimens in the Greater Mekong Subregion (GMS) to review clinical and molecular epidemiology in both nosocomial and community settings, the diagnostic methods, and the risk factors. The annual prevalence of ESBL-*Escherichia coli* (ESBL-Ec) increased in all clinical samples, especially in clinical blood samples. The study also found that ESBL is significantly associated with recent exposure to broad-spectrum antibiotics, presence of comorbidities, and chronic kidney disease. Phenotypic ESBL was detected by double disk synergy test, E test, and VITEK-2, whereas carbapenemase was detected using Modified Hodge test or mCIM method. PCR was the most common genomic detection method, followed by WGS and a combination of both.

The topic focused on not only clinic but also veterinary environments. Elbediwi et al. highlighted the advanced approach, genomic characterization, and statistical analysis in routinely monitoring the emerging AMR *Salmonella enterica* circulating in breeding chicken hatcheries in Henan province. The findings also proposed that one of the potential pathways for the spread of MDR *Salmonella enterica* serovars could be the day-old breeder chicks' trades.

A sudden appearance of emerging MDR *Candida auris* capable of rapid transmission is under close attention. Hu et al. conducted a retrospective analysis of *Candida auris* infection cases up until December 31, 2020 in PubMed and Web of Science databases to determine the risk factors on patient mortality. The study revealed a higher proportion of men, premature babies, and elderly people in cases. The proportions of patients with diabetes, kidney disease, trauma, or ear disease were high. More than half of patients had a history of central venous catheter use and broad-spectrum antibiotic use. The study revealed that only kidney disease was an important risk factor for mortality in *Candida auris*-infected patients.

In conclusion, MDR pathogens is an ever-changing problem that requires systematic research and long-term monitoring to limit the further spread of resistant bacteria. Currently, advanced bioinformatic technologies, including whole-genome sequencing, phylogenetic network analysis, and cluster analysis, have promoted research on the evolution of MDR pathogens, even the hypervirulent and MDR pathogens. The development of better detection and typing methods needs further exploration.

## Author Contributions

YZ and QY wrote the article. Both authors contributed to the article and approved the submitted version.

## Funding

This study was sponsored by funding from the National Natural Science Foundation of China (No. 82072318) and National Key Research and Development Program of China (2018YFE0101800 and 2021YFC2301002).

## Conflict of Interest

The authors declare that the research was conducted in the absence of any commercial or financial relationships that could be construed as a potential conflict of interest.

## Publisher's Note

All claims expressed in this article are solely those of the authors and do not necessarily represent those of their affiliated organizations, or those of the publisher, the editors and the reviewers. Any product that may be evaluated in this article, or claim that may be made by its manufacturer, is not guaranteed or endorsed by the publisher.
